# The Caring Mission – Nursing Personnel’s Inner Driving Force in End-of-Life Care

**DOI:** 10.1177/23333936221128241

**Published:** 2022-10-31

**Authors:** Margareta Karlsson, Christina Karlsson, Anne Kasén

**Affiliations:** 1 University West, Trollhättan, Sweden; 2 Specialized University, Bergen, Norway

**Keywords:** caring, driving force, end of life, nursing personnel, mission, Sweden, drivkraft, livets slut, mission, Sverige, vårdandet, vårdare

## Abstract

Constantly facing human suffering and impending death can generate anxiety and insecurity in nursing personnel in end-of-life care. The aim of the study is to reveal nursing personnel’s inner driving force in end-of-life care. A phenomenological hermeneutical method was used to search for meaning in the narrative data collected in this study. The structural analysis resulted in four themes: *The appeal in the patient’s vulnerability, The appeal in the patient’s joy, Facing one’s own existence in vulnerability*, and *Being at home with colleagues*. Both vulnerability and joy motivated nursing personnel in caring. The care was often emotionally engaging and oscillated between grief and joy, which required a great deal from the nursing personnel both as professionals and fellow human beings. At the same time the emotionally engaging constituted an inner driving force, which gave them courage to do the best for the patients at the end of life.

## Swedish Abstract

Att ständigt möta mänskligt lidande och förestående död kan skapa ångest och osäkerhet hos vårdare som vårdar patienter i livets slutskede. Syftet med studien är att synliggöra vårdares inre drivkraft vid vård i livets slutskede. En fenomenologisk hermeneutisk metod användes för att söka efter mening i vårdarnas berättelser. Strukturanalysen resulterade i fyra teman: *Appellen i patientens sårbarhet, Appellen i patientens glädje, Att möta sin egen existens i sårbarhet*, och *Att vara hemma med kollegor*. Både sårbarhet och glädje motiverade vårdarna i vårdandet. Vårdandet var ofta känslomässigt engagerande och vårdarna pendlade mellan sorg och glädje, vilket krävde mycket av dem både som yrkesmänniskor och medmänniskor. Samtidigt utgjorde det känslomässiga engagemanget en inre drivkraft, som gav dem mod att göra det bästa för patienterna i livets slutskede.

## Introduction

Caring for patients in a spirit of caritas is demanding, thus nursing personnel need courage to care, a courage grounded in their own existence (Eriksson, 2018). In particular, caring for patients in end-of-life care can be demanding as the nurses become deeply involved in the patients’ and relatives’ existential questions (Fay & O’Boyle, 2019). Consequently, nurses’ impressions of shortcomings due to lack of time (Holst et al., 2003; Sørlie et al., 2004) and feelings of helplessness as professionals when failing to alleviate the patient’s spiritual and existential suffering have been reported (Tornøe et al., 2015). Nurses can also be confronted with ethical dilemmas in the care of the patients and their relatives (Karlsson et al., 2010, 2015), while their own existential questions about life, suffering and death can also arise. Such existential questions can affect them in their becoming as nurses (Karlsson & Kasén, 2021).

Becoming as a nurse can be characterized as being in a continual movement and may result in awareness of death, give meaning to their lives and add to their understanding of life and existence (Nyström, 2014). Caring for patients in end-of-life care seems to deepen nurses’ contact with their own existential questions, which can strengthen their understanding of becoming as a human being (Karlsson & Kasén, 2021).

In a study about caring for dying patients, Tornøe et al. (2015) found that the fulfilment the nurses occasionally felt when managing to help patients achieve reconciliation counterbalanced other common challenges and feelings of shortcomings associated with nursing care. Likewise, studies in other contexts also show that practice based on inner core beliefs makes nursing meaningful and is a central aspect in the decision to remain in nursing despite challenges (Dunn, 2012). The nurses in end-of-life care described how they want to help patients; they see and understand patients’ suffering, take responsibility and have compassion for them, in addition to doing their utmost to fulfill the patients’ wishes and enhance their wellbeing (Karlsson et al., 2015). Therefore, the focus of our inquiry is the phenomenon, the “what,” that fuels the nurses’ engagement, their sense of responsibility and their care for this group of patients.

We approach the question from what Eriksson (2018) wrote about nurses caring in love. She noted that nurses also emanate the force and light of love, “caritas” and “claritas,” symbolizing the core of caring science. If a person does not want to fulfill a task, the work will not be done with joy or enthusiasm (Eriksson, 2018). Kierkegaard (1862/2011) also pointed out that charity is a deed of love, true and beautiful. Charity is made visible in *how* help is given, not in *what* is given.

Nurses’ attitude to caring for the patients and their relatives is essential in end-of-life care (Karlsson et al., 2013; Mahiro et al., 2014). Nursing care staff in Young et al.’s (2017) study described advocating, caring, communicating, and relating as values of importance that have an impact on a good death in end-of-life care. Hospital nurses go from one room to another between life and death but feel grateful to be able to share the end of another’s life and strive to care for both patients and their relatives in the best possible way (Johansson & Lindahl, 2012). Nurses experienced challenges providing emotional and psychological support to patients who were at the end of life and their families in a surgical setting (Limbu & Taylor, 2021). Nurses who care for dying patients outside specialist palliative care settings are described as being both determined to provide high quality care and at the same time disappointed because of the lack of time and resources. However, relationships with patients and relatives, cooperation with colleagues and the possibility for personal growth seem to contribute to satisfaction (Wallerstedt & Andershed, 2007). The physical surroundings can also contribute to nurses’ sense of wellbeing, as Johansson and Lindahl (2012) described how a specially designed patient room for end-of-life care meant a great deal to all involved. It improved the quality of care and the nurses felt more comfortable, satisfied, and secure.

Ward et al. (2021) show that family carers can be emotionally affected when caring for patients who are dying at home, but with the support of a hospice-at-home service their wishes can be fulfilled. It also highlights the importance of working together with family carers and patients (Ward et al., 2021).

Constantly facing human suffering and impending death can generate anxiety and insecurity both in the team surrounding the patient and in the care. How can nursing personnel find the driving force in caring for patients in end-of-life care? The aim of this study is to reveal nursing personnel’s inner driving force in end-of-life care.

## Method

In line with the aim, a qualitative inductive approach was selected as the research design. We applied a phenomenological hermeneutical method (Lindseth & Norberg, 2004, 2022) in order to illuminate and reflect on nursing personnel’s lived experiences of the inner driving force in caring for patients in end-of-life care. This method is inspired by Ricœur’s philosophy, but does not provide a recipe for conducting the analysis instead serving as a recommendation for investigating and disclosing essential meanings of life (Lindseth & Norberg, 2022). Singsuriya (2015) also contends that Ricoeur never created a method but presented a theory of interpretation, where the hermeneutic arc of explanation and understanding is central. This hermeneutic arc is present in the three steps of the phenomenological hermeneutical interpretation. Together, these three steps constitute a hermeneutic circle (Ricoeur, 1976).

Phenomenological hermeneutical interpretation in accordance with Lindseth and Norberg (2004) guides the researcher through the three steps. The first step is an initial naïve reading of the whole text to gain a naive understanding of nursing personnel’s inner driving force in end-of-life care. The second step in the phenomenological hermeneutical interpretation consists of a structural analysis. In the structural analysis the text divides into meaning units, condensed, and abstracted into sub-themes and themes. In this step it is important to stay close to the material and compare it with the naive understanding before finally returning to the whole of the text through a comprehensive understanding of the naive understanding and the themes (Lindseth & Norberg, 2004).

### Study Design and Participants

Data were collected at a palliative care unit in Sweden as part of a larger project about nursing personnel’s personal and professional development for a sustainable working life in end-of-life care. This is the first study in a series from this piece of research.

The participants in the project were assistant nurses (unlicensed assistive personnel) and registered nurses (RN) with work experience from a palliative care unit and willing to participate in the study. In Sweden, assistant nurses provide practical nursing to patients in, for example, primary healthcare and municipal care. Initially, nine participants were included in the study, but two of the audiotaped interviews were inaudible and could not be used, resulting in a total of seven interviews, with six RNs and one assistant nurse. The participants were women aged between 25 and 65 years, with a median age of 42 years. Their work experience in the palliative care unit varied between 1.5 and 11 years. The participants are presented in a general manner to preserve their anonymity. In the presentation the participants are referred to as nursing personnel.

### Data Collection

The second author who performed the interviews at the participants’ workplace strove to obtain narrative interviews, where her pre-understanding was challenged by the horizon of understanding from the participants’ perspectives and possible misunderstandings were clarified (cf. Lindseth & Norberg, 2004). In order to capture narratives about their life world as nurses in end-of-life care we also asked them to describe their workday, and what creates meaning in their own lives. During the interview the second author asked further questions such as: What do you mean, Can you clarify that, What were your feelings. When the participants narrate their lived experiences of the phenomenon, we as authors can search for the meaning of nursing personnel’s inner driving force in end-of-life care (cf. Lindseth & Norberg, 2022). The interviews, which were audiotaped and later transcribed verbatim, were conducted at the participants’ workplace in spring 2019 and generated rich data material.

### Analysis and Interpretation

In order to gain as truthful an interpretation as possible of the meanings embedded in the narrated experiences, we strictly adhered to Lindseth and Norberg’s (2004) description of the phenomenological hermeneutical method. All authors analyzed the text together in a dialogical process during three nonlinear phases, moving between the parts and the whole. During the naive reading we focused on grasping the meaning of the nursing personnel’s experiences of end-of-life care in the palliative care unit. In the structural analysis we strived to stay as close as possible to the material. Here our material was divided into meaning units, then condensed, abstracted and formulated into sub-themes and themes. The first immediate understanding of the meaning in the nursing personnel’s experiences from the initial, naïve reading was thereby validated against the explanation from the structural analysis, a “test” not of correctness, but about the meaning in lived experience. For us, this “test” resulted in a new naïve reading, which in turn gave us a reason to revise the structural analysis, a process that created a hermeneutical, circular movement of understanding as recommended by Lindseth and Norberg (2022). Finally, in the third phase we critically reflected on the naive understanding, themes and sub-themes. According to Lindseth and Norberg (2004), comprehensive understanding means that the text is read as a whole, including the naïve understanding and the themes.

### Rigor

Trustworthiness in phenomenological hermeneutical studies is challenging, as each study is unique and no repetitive process is either desirable or possible. The search is not for a “single fundamental truth,” but for possible meanings (Lindseth & Norberg, 2004, p. 151). To ensure the validity of the study and the interpretation of the data we as the authors were aware of our pre-understanding of the phenomenon and strived to take as objective an approach as possible to the interpretation during the naïve understanding, structural analysis and comprehensive understanding. Nevertheless, our understanding of the material is colored by our pre-understanding. The main themes and sub-themes that emerged in the structural analysis are reflected on in relation to the research context, research questions and the naïve understanding. During the interpretation the authors discussed the naïve understanding, the structural analysis and the comprehensive understanding to reveal nursing personnel’s inner driving force in end-of-life care. This is consistent with Lindseth and Norberg’s (2004, 2017) phenomenological hermeneutical research.

### Ethical Considerations

The Regional Ethical Review Board (Dnr: 1161-18/2019-00634) approved the study. Participants received written and oral information about the aim of the project. The nurse manager at the palliative care unit informed about the study, provided written information and asked if there were any nursing personnel who were interested in voluntarily participating in the study. The written information about the study, stated that participation was voluntary and that they could withdraw at any time without giving a reason. The data were treated according to the principles of Research Ethics Delegation (The Finnish Advisory Board on Research Integrity (2012) and the World Medical Association (2013) Declaration of Helsinki. All the data was de-identified and stored in such a way that no unauthorized persons can access the material. Participants’ personal data were handled in accordance with the General Data Protection Regulation (GDPR 2016/679).

### Interpretation Process and Findings

In this section the interpretation process and findings are described. The naïve understanding is first presented to gain an initial understanding of the whole, after which the four themes that emerged in the structural analysis are illustrated and finally a comprehensive understanding and reflections are illuminated.

### Naïve Understanding

The nursing personnel face complex situations when caring for patients at the end of life, but they described joy at being able to help and make a difference so that the patients’ final weeks can be beautiful both for themselves and their relatives. The nursing personnel felt privileged to be with the patients during their last days of life. The conversations, closeness and being present with the patients and their relatives seem meaningful for the nursing personnel in end-of-life care.

The nursing personnel felt empowered when they could make a difference for dying patients and their relatives and contribute to making patients’ end of life peaceful. They stated that when patients are at peace with their situation it gives them strength.

It was not the big issues in nursing care that made the nursing personnel feel they were doing good. Caring is not always a question of following routines. On the contrary, caring in end-of-life care may involve abandoning routines in order to do what is best for the patient. The nursing personnel dared to question routines and derived strength from seeing how satisfied the patients and their relatives were.

The well-being they felt together with their colleagues at the palliative care unit also gave them strength. The work culture at the unit is tolerant and they can support each other. Grief can also be brought to the team and managed together. Sharing feelings and discussing them with others strengthened the nursing personnel.

The nursing personnel also pointed out that caring for patients in end-of-life care can be both physically and mentally burdensome and it is therefore important to listen to one’s own body. One needs the strength gained from meaningful leisure time, healthy eating, exercise, sufficient sleep and feeling loved and confirmed. The nursing personnel contended that awareness of the finiteness of life also contributed to a deep gratitude for their own life and well-being. They considered it essential to discuss life and death, emphasizing their insight into the importance of taking care of life, living it to the full and not taking things for granted.

### Structural Analysis

In the structural analysis four themes emerged as a source of strength for the nursing personnel in end-of-life care. For an example of the structural analysis process see Table 1. The themes are; *The appeal in the patient’s vulnerability, The appeal in the patient’s joy, Facing one’s own existence in vulnerability*, and *Being at home with colleagues*. Both vulnerability and joy appealed to the nursing personnel and awakened their mission to help. They described how the conversations about existential questions of life and death with patients and their relatives strengthened them. Feeling at home in the workplace was important, while feeling safe with colleagues and being able to be oneself also strengthened them.

**Table 1.** Example of the Structural Analysis Process.

Statement	Condensed statement	Sub-theme	Theme
They are terminally ill and I really want them to have the best care possible. If it is difficult, it motivates me a little to walk away from the routines. Make a kind of detour routine (IP1)	They are terminally ill and I want to do good and that motivates me to abandon routines. It gives me so much strength to keep fighting.	Being touched by the patient’s vulnerability	The appeal in the patient’s vulnerability
To see the joy in the patient’s eyes when seeing her/his dog as they may not have seen each other for a week. It gives me so much strength to fight on. (IP1)	Seeing the joy in the patient’s eyes gives me (as a healthcare professional) such power to fight on.	Being touched by the patient’s joy	The appeal in the patient’s joy
Sometimes it’s tough when you identify yourself with a human being who is at the end of life, where you can transfer the situation (IP8)	Identifying oneself with a human being who is at the end of life	Facing one’s own existence	Facing one’s own existence in vulnerability
It feels safe here, like a family. It feels like this is my home and the human beings. . . (IP5)	Feels like home at work	Feeling of home at work	Being at home with colleagues

### The Appeal in the Patient’s Vulnerability

A driving force for caring in end-of-life care is the patients’ vulnerability, which appeals to the nursing personnel and triggers compassion with the patients. The patients’ vulnerability touched the nursing personnel and made them willing to fight to alleviate the patients’ suffering. However, they sometimes experienced despair, frustration, sadness and anger as the problems were complex and difficult to alleviate.

Despite the difficulties in the nursing care itself and irrespective of the above-mentioned negative feelings, the nursing personnel felt privileged to be part of the caring and wanted to do their best for the patient during her/his last days of life. One participant described how the patient’s vulnerability appealed to her and gave her strength to care:

*If it’s a patient you can relate to, who’s either younger than me. . . or is. . . maybe of my own age or a few years older. Well, then the brain starts working, because then I usually think oh, this could be me. . .* . . . *and that is what triggers me most, these young patients. . . . .* (IP4)

Situations that participants could relate to their own life affected them emotionally. However, they could talk about it together in the team and gain different perspectives on the situation, which strengthened them as nursing personnel.

The nursing personnel gained strength when they felt that they could help to make the situation better for patients and their loved ones at the end of life. Even if the patients could not be cured, the nursing personnel could fight to ensure that the end of life was good and thus an experience of health. One of the participants describes the power of care: *I feel like I’m gaining strength when I can help to make the situation better, even if the person doesn’t become healthier. . .* (IP1)

Being able to give something of themselves, both as nursing professionals and human beings, contributed strength to fight for the patients at the end of life. They were interested in the patients as human beings and looked beyond the disease. In order to provide individual care, it was motivating to deviate from routines and focus on the patients’ needs as human beings. Below, a participant describes departing from routines:

*. . . and just abandoning routines, I think, constitutes palliative care. It’s like a book that hasn’t been written yet, and then you might think that you should make the best of the situation. Maybe get away a little from the routine and despite having to face the consequences afterwards. . . and it motivates me a little to get away from routines sometimes* (IP1)

For example, abandoning routines could involve arranging for the patient to meet her/his dog because the nursing personnel knew that it meant so much for the patient’s well-being, even though it was not allowed on the unit.

### The Appeal in the Patient’s Joy

When the patients experienced well-being at the end of life, the nursing personnel could see how they radiated joy. In such situations, the nursing personnel saw how they could make a difference for patients and their loved ones in the difficult circumstances that the end of life entailed. The patients’ joy appealed to the nursing personnel. Being able to help the patient at the end of life was perceived as a privilege and gave meaning to the nursing personnel’s own lives. One participant describes how she felt when she saw the patient’s joy: *Seeing the joy in the patient’s eyes gives me (as a nursing professional) such strength to fight on* (IP1) The patients’ joy was a driving force that enabled the nursing personnel to continue to fight to relieve patients’ suffering. Creating a caring relationship with patients and their loved ones gave strength to care and a special sense of presence in the room.

The patients treated at the palliative care unit had complex needs. When the nursing personnel felt that their quest to relieve patients’ suffering contributed to a sense of wholeness and well-being, they experienced an inner feeling of having succeeded as a team. A participant described the feeling as follows: *You’re happy when you walk away (from the palliative care unit) and feel like “God what a great team job we’ve done” when we’ve succeeded, when we’ve relieved someone, because we have very complex patients. . .* (IP4)

Although the patients had complex problems that were difficult to alleviate, it was a good feeling when the nursing personnel felt that they had eased the patients’ suffering. Two participants describe the experience of meaningfulness in the care: *It’s such a bad symptom and so. . . But it is also very positive when you feel that you can alleviate it* (IP7) *. . . I think it’s so nice being able to influence the end of life and make it good. I often see it as a privilege to be present and participate on the last journey* (IP8). In addition to being able to relieve complex symptoms, making a day meaningful, meeting patients who were calm and had reconciled with life and their situation, as well as supporting the patients’ loved ones gave them strength.

It was important to help and support both the patients who were dying and their relatives who would live with the memory of these last days for the rest of their lives. The nursing personnel had many meetings with patients and relatives and a range of feelings arose in this difficult situation, but mainly joy and love. One participant stated:

*There are many meetings and a lot of feelings and a lot of love and a lot of joy. . . well really much more love and joy than grief. Many people think that it is all about death and grief, but there’s so much more.* (IP9)

The love and joy that appealed to the nursing personnel were a driving force in their work. It was important for the nursing personnel that others should understand that end-of-life care is not only about death and grief, but also contains a lot of joy. The nursing personnel also experienced that the patients gave them so much back and the good relationships they had with their colleagues strengthened their inner driving force in caring.

### Meeting One’s Own Existence in Vulnerability

The driving force in caring for patients at the end of life also involved meetings with patients and relatives on existential issues about life and death. There was also a meeting in the nursing personnel’s own vulnerable inner rooms, with thoughts and questions about their own existence, life and death. Meetings with patients and relatives about existential issues meant that nursing personnel developed a deeper relationship and got to know the patients as human beings:

*. . .so are there some to whom you are closer. . . I usually report on the patient when the next work shift arrives, but I have gone and said goodbye to. . . when I am free over the weekend and understand that they will no longer be here. . . I do not know if it was selfish because it was for my own sake that I said goodbye. . . but I felt that way, in these meetings. But otherwise, I usually try not to take that home with me.* (IP6)

The most difficult encounters were those with patients and loved ones who had young children or patients who were the same age as or even younger than their own children. The participants described the encounter with their own existence in such a situation: *We had someone the same age as my son. Of course, it’s going to be a little like that. Or if they have young children or maybe they’re my own age. . .* (IP6). *. . . sometimes it’s tough when you can identify with a human being who is at the end of life, where you can transfer the situation* (IP8). The nursing personnel considered that they worked with life and that life was in focus, hence it was important to be able to help the patients to live until the end. Being able to be involved and help patients to have as good a time in life as possible was a source of power. One participant described how the care focuses on life: *. . . We are actually working with life. You live until you die. We don’t just work with death.* (IP6). Focusing on life during patients’ last days gave the work meaning. This meant focusing on relieving the patient’s total suffering in body, soul and spirit. Another participant described what gives meaning to the work: *I feel that I am constantly being challenged and growing both as a human being and as a nurse. . . It’s important for me.* (IP9)

Being challenged in caring gave the nursing personnel strength and they struggled together in the team to do their best for the patients and relatives. It made the work meaningful when the nursing personnel felt they had succeeded. This was counterbalanced when they felt that they were unable to relieve the suffering or when the team members did not agree about the care of the patients. When they disagreed with the team, it could be that the doctor did not see what the other nursing personnel saw, for example continuing treatment although the patient was dying. Below, one participant describes this frustration: *. . . Sometimes you feel frustrated when the doctor doesn’t see what we see. A patient who is dying and the doctor continues. . .* (IP7)

End-of-life care was compared to maternity care. You are born once and die once, hence the nursing personnel felt that they had only one chance to ensure a dignified experience for patients and their relatives. It was essential for the relatives, who must live with the experience for the rest of their lives. One participant describes how end-of-life care can be compared to maternity care:

*Compare it to childbirth. You only give birth to this child once and you want to make it a positive experience. And I feel the same when you die, you only die once and the relatives and yes, partly the patient her/himself, should have as good a death as possible because those relatives will carry the experience with them. . .* (IP6)

### Being at Home With Colleagues

Love and respect between colleagues were a driving force. The tolerance level was high, which was considered necessary to cope with the work of the palliative care unit. The nursing personnel felt that as colleagues they could listen to and confirm each other, which evoked a sense of being at home even when they were at work. Below, a participant expressed: *We have a very high tolerance level on the unit. . . You must have that to work in a unit like this. There is a lot of love at work. We are pretty good at confirming each other* (IP8). The nursing personnel did not always agree on what happened or what to do in difficult and complex situations. But there was a sense of security in being allowed to express what they saw, thought and felt in complex situations that were difficult to handle.

The nursing personnel derived meaning from going to work when they felt that they were respected by colleagues. It gave them the impression that everyone was equally important in the care of the patients, regardless of their professional category. The cohesion of the team and the work with different professions were fundamental in making the situation as good as possible for patients and relatives. One participant described the feeling of being at home on the palliative care unit: *. . . I love coming here. I always look forward to going to work. . .* (IP5).

The nursing personnel perceived palliative care as a calling, not just a job. They felt called to care for patients at the end of life because they wanted to make a difference and had the strength to help patients and their relatives. One participant described the care as a vocation: *I see this as some kind of calling. . . like I’m called to service. It’s not just a normal job for me* (IP7). The nursing personnel’s sense of being at home in the palliative care unit was due to the fact that they enjoyed themselves and had cohesion and openness, where there was room for both laughter and tears. It was important to be able to show emotions and have the opportunity to talk about them when complex situations became too difficult mentally. One participant described the cohesion: *It’s not just laughter, you have to cry too. Don’t be afraid of showing emotions* (IP1). Showing emotions in difficult and complex situations was a way of dealing with the circumstances and being allowed to do so provided a sense of security for the nursing personnel. In these situations, the nursing personnel were there for each other to confirm and show compassion for each other as human beings, which gives strength in caring. Laughing and crying together were also a source of the driving force.

The nursing personnel enjoyed their work and looked forward to working on weekends. It was not because there was little to do, but because going to work was like coming home. The nursing personnel felt at home on the unit and could not imagine working anywhere else. When their working day was over, they were able to leave work with the feeling that they had strived to do good for patients and their relatives at the end of life.

### Comprehensive Understanding and Reflections

The structural analysis showed that patients’ vulnerability and joy appeal to the nursing personnel and awaken their compassion, thus giving them strength to provide end-of-life care. The vulnerability aroused compassion to continue the struggle to relieve the patients’ suffering. The joy that appealed to the nursing personnel aroused their compassion when they saw how they could make differences in caring and alleviate patients’ suffering in the difficult and complex situations they found themselves in. This can be compared to the fact that Karlsson and Kasén (2021) believe that nursing personnel in the caring community develop deeper contact with their own lives as human beings, a movement inwards to consciousness and ethos. In life as human beings there is both sadness and joy that nursing personnel have to become reconciled with (Karlsson & Kasén, 2021). When nursing personnel met patients and relatives on issues related to life and death they faced their own vulnerability and thoughts about their own life and death. It was a meeting in a spatial vulnerability and contributed to deepening the relationship with the patient and relatives, which facilitated the care as it helped them to understand the patient’s needs. Karlsson and Kasén (2021) believe that love makes nursing personnel vulnerable and receptive to the inner voice of wanting to help, which awakens their compassion to help the patient in a selfless and loving way. The encounter with one’s own existence in vulnerability when caring at the end of life was compared with the care at the birth of a child. Human beings are born only once and so it is with death too, every birth and death are unique. Caring for patients at the end of life is also a unique experience for healthcare personnel. Nursing personnel’s feeling of being at home with their colleagues contributes to a sense of security and being at home in themselves. It becomes a home in the collegial space where care is based on an ethos where the human being feels at home and where there is love that gives a driving force in the care of patients at the end of life. The feeling of being at home can be understood from the theoretical model presented by Karlsson and Kasén (2021), where nursing personnel in a communion gain contact with their own lives and life situations as human beings. Nursing personnel felt that they could leave work at the end of the working day with an inner feeling that they had done good in caring for the patients and their relatives. This can be compared to what Karlsson and Kasén (2021) describe as the endless guilt of love for the other, which can provide the inner driving force and the joy of wanting to help in a selfless way.

## Discussion

The study findings indicate that patients’ vulnerability and joy appeal to the nursing personnel’s sensitive inner room both as healthcare professionals and as human beings when caring for patients at the end of life. A source of the driving force in caring is thus that patients’ vulnerability but also their joy appeals to the nursing personnel, motivating them to struggle to relieve suffering and enabling them to see and understand that they can help and make a difference. This gives strength to nursing personnel in end-of-life care and can be compared with Tornøe et al. (2015), who found that nurses who help patients achieve reconciliation eliminated other common challenges and shortcomings associated with nursing care.

Caring for patients and their relatives at the end of life is also a meeting in the nursing personnel’s own vulnerability as human beings and their own existential situations. This is in line with Angel and Vatne (2017), who state that the core of vulnerability concerns the endeavor of patients and nurses in their becoming as persons. Nurses’ own vulnerability is exposed in the encounter with patients, in which nurses’ can feel a threat to their existence if they fail to provide good nursing when caring for patients (Angel & Vatne, 2017). It is difficult to distinguish between nurses’ vulnerability in the professional and the private spheres (Angel et al., 2020).

Feeling at home on the unit can promote nursing personnel’s becoming as human beings. The nursing personnel’s inner ethos is awakened in the caring communion of patient vulnerability and joy, which contributes to becoming by providing a deeper understanding of health, suffering, life, and death. This is in line with Karlsson et al. (2017), who revealed that nurses in caring communion at the end of life became aware of their own existence as fellow human beings.

The sense of being at home with colleagues experienced by the nursing personnel can facilitate becoming as a human being and as a professional, providing an inner driving force in the care of patients at the end of life. The sense of being at home also consisted of the nursing personnel feeling secure with their colleagues and seeing the care of patients at the end of life as a calling, and not just a job to earn money. They felt they were in the right place in life and that they could make a difference. This can be compared to how Eriksson (2018) describes the call as a personal and ethical position. Being called should not be confused with being subordinate and not demanding a salary for the work but equated with love that provides the driving force, where the basic attitude comprises generosity and joy (Eriksson, 2018). The driving force can also be understood from Kierkegaard (1862/2011) point of view that charity is made visible in *how* help is given, not in *what* is given.

Emerson (2017) analyzed the concept of a vocation to nursing and found both negative and positive consequences. Among the positive consequences were meaningfulness and engagement in the work but also personal wellbeing and satisfaction. One of the negative consequences was that it could lead to problems with the work-life balance. Emerson (2017) defines the calling to nursing as follows: *a calling is a passionate intrinsic motivation or desire to help others through engagement in nursing practice as a means of giving purpose to one’s life* (p. 4).

This is in line with Eriksson’s (2018) statement that it takes courage to dare to be called and strive to do what deep down feels right (Eriksson, 2018). Afsar et al. (2019) contended that a vocation can give nurses a higher degree of satisfaction and engagement in nursing, making them more willing to stay within the organization. This confirms the result of the present study about nursing personnel’s experiences of being at home at work and the security it provided. This is also in line with Dunn’s (2012) study, in which it is stated that inner core beliefs are meaningful and a central reason for remaining in nursing despite challenges. Ablett and Jones (2007) revealed that nurses working in a hospice were satisfied with their career choice, especially palliative care. Awareness of their own mortality gave them zest for life, while awareness about their own spirituality helped them in providing care for patients and their families. Job satisfaction depended on support from colleagues, time to listen and talk to the patients and a pleasant environment at work (Ablett & Jones, 2007). In a study of palliative rehabilitation, Gamskjaer et al. (2022) found that collegial feedback and having a balance in working life was important for healthcare professionals’ job satisfaction. Healthcare professionals also experienced that the work was meaningful, but it could sometimes occupy their minds, making it difficult to manage their own home and family situation (Gamskjaer et al., 2022). That palliative care has an emotional impact on personnel as human beings also emerged in our study.

The care was often emotionally engaging and oscillated between grief and joy, which demanded a great deal from the nursing personnel both as professionals and as fellow human beings, while at the same time it constituted an inner driving force for caring. The study by Rivera-Romero et al. (2019) shows that nursing personnel in an intensive care unit have a range of feelings in their relationship with the patients’ family in end-of-life care. The feelings consist of sadness, pain and impotence. The nursing personnel wanted to be strong when supporting the families, so tried to not cry. However, there was a need for spaces where they could express their feelings alone (Rivera-Romero et al., 2019). Our study revealed that emotionally engaging and oscillating between grief and joy is a driving force that can give nursing personnel the courage to do their best for patients who are at the end of their lives. However, to ensure a sustainable working life, nursing personnel may need support to manage their emotional engagement and oscillation between grief and joy and how it can affect them.

Liu and Chiang (2017) show that life and death issues and witnessing patients suffering in end-of-life care is an opportunity for nurses to face their own inner selves. In addition, they reported that some nurses will quit their work in end-of-life care while others will stay. *The nurses who decided to stay in end-of-life care had formed new attitudes or states of mind consisting of courage, calmness and passion* (Liu & Chiang, 2017, p. 33). When Diehl et al. (2021) compared general and specialized palliative care nurses, they found that specialized palliative care nurses had a better health status than general palliative care nurses. Specialized palliative care nurses were more likely than generalized palliative care nurses to stay in the profession, although there were no differences regarding engagement at the workplace and satisfaction with life (Diehl et al., 2021).

Where there is a sensibility to patients’ vulnerability and an inner flow of selfless love, end-of-life care can bring joy, strength and courage to caring, as well as understanding and meaning to the nursing personnel’s own lives. The caring mission as a driving force can contribute to the movement in becoming both as a professional and as a human being.

### Limitations

This is the first study in a larger project and is based on participants from a palliative care unit in order to gain nursing personnel’s lived experiences of the meaning of inner driving force. The data consist of seven interviews, which is in line with the focus on depth and meaning in the phenomenological hermeneutical approach. We contend that the substance in the seven interviews was sufficiently rich to fulfill the aim of the study. A limitation may be the fact that only personnel from a palliative care unit were included, but in order to gain nursing personnel’s lived experience of inner driving force in end-of-life care, it is essential to interview nursing personnel who only care for patients at the end of life.

## Conclusion and Implications

This study may contribute to helping nursing personnel to gain a deeper understanding of the inner driving force in end-of-life care. Nursing personnel’s inner driving force in end-of-life care consists of the patients’ appeal of vulnerability and joy, as well as nursing personnel’s meeting with their own vulnerability and the feeling of being at home with colleagues. The findings of the present study may also help nurse managers appreciate how patients’ emotions appeal to nursing personnel and that nursing personnel may require support for a sustainable working life. Nursing personnel’s inner driving force in end-of-life care needs to be highlighted. If nursing personnel can become aware of and affirm their inner driving force it can give them courage to do what is true, good and beautiful for patients at the end of life.
