# Corrigendum to The Caring Mission—Healthcare Personnel’s Inner Driving Force in End-of-Life Care

**DOI:** 10.1177/23333936221144938

**Published:** 2022-12-01

Karlsson, M., Karlsson, C., and Kasén, A. (2022). The Caring Mission – Healthcare Personnel’s Inner Driving Force in End-of-Life Care. *Global Qualitative Nursing Research*. https://doi.org/10.1177/23333936221128241

The authors would like to notify that since the original online publication, the article title has been changed from “The Caring Mission—Healthcare Personnel’s Inner Driving Force in End-of-Life Care” to “The Caring Mission – Nursing Personnel’s Inner Driving Force in End-of-Life Care”.

